# Comparison of four nutritional screening tools in perioperative elderly patients: Taking orthopedic and neurosurgical patients as examples

**DOI:** 10.3389/fnut.2023.1081956

**Published:** 2023-03-29

**Authors:** Jie Gong, Silu Zuo, Jie Zhang, Li Li, Jie Yin, XinYi Li, Fengmei Yu, Wen Hu

**Affiliations:** ^1^Department of Clinical Nutrition, West China Hospital, Sichuan University, Chengdu, China; ^2^Department of Orthopedics, West China Hospital, Sichuan University, Chengdu, China; ^3^Department of Neurosurgery, West China Hospital, Sichuan University, Chengdu, China

**Keywords:** nutritional screening tools, nutritional risk, perioperative, elderly, prognosis

## Abstract

**Background and aims:**

Malnutrition is widely present in elderly surgical patients and is highly correlated with prognosis after surgery. However, studies comparing the effectiveness of comprehensive nutritional screening tools in geriatric surgical patients have not yet been published. The nutritional risk among elderly orthopedic and neurosurgical patients and their associated clinical indicators and outcomes was assessed using four screening tools. The aim of this study was to explore suitable tools for screening the nutritional status and identify their potential to act as prognostic indicators.

**Methods:**

The Nutritional Risk Score 2002 (NRS2002), Mini Nutritional Assessment - Short Form (MNA-SF), Geriatric Nutritional Risk Index (GNRI), and Prognostic Nutritional Index (PNI) were all performed within two days of admission and before surgery. The relationships between nutritional risk classifications and conventional nutritional markers, complications and length of hospital stay (LOS) were evaluated.

**Results:**

In this study, a total of 167 orthopedic patients and 103 neurosurgical patients were evaluated. In neurosurgical patients, the rates of malnutrition or patients at risk of malnutrition according to the MNA-SF, GNRI, NRS2002 and PNI were 26.4, 24.6, 8.4, and 12.6%, respectively. According to the NRS2002 and PNI, the rates of old neurosurgical patients who were malnourished or at risk of malnutrition were 14.6 and 3.9%, respectively, which were lower than the results assessed by the MNA-SF (24.3%) and GNRI (15.5%). Multiple regression analysis revealed a significant relationship between the PNI (malnourished vs.well-nourished, OR = 5.39, 95% CI:1.11-26.18, P = 0.037), GNRI (at risk vs.no risk, OR = 3.96, 95% CI: 1.01-15.45, *P* = 0.048) and the complications in orthopedic patients. Only GNRI was significantly related to LOS > 7 days (at risk vs.no risk, OR = 4.01, 95% CI: 1.64-9.80, *P* = 0.002). For neurosurgical patients, an association between GNRI and LOS > 8 days was discovered (at risk vs.no risk, OR = 3.35, 95% CI: 1.03-10.86, *P* = 0.002).

**Conclusion:**

Among the four nutritional risk screening tools, the GNRI exhibited better predictive value for short-term outcomes in elderly perioperative orthopedic and neurosurgical patients, thereby suggesting that it might be a more suitable tool for nutritional risk screening. Additional studies are required to determine the applicability of GNRI in other surgical fields.

## 1. Introduction

The percentage of elderly patients undergoing surgery is progressively increasing with the acceleration of aging. Previous studies have shown that the prevalence of malnutrition in elderly surgical patients is as high as 41.6%, and the presence of nutritional risk accounts for 20.8%, which are both significantly compared to young and middle-aged patients (1).

Furthermore, malnutrition is highly correlated with the prognosis of elderly perioperative patients, which can result in poor clinical outcomes, increased morbidity and mortality, complication rates, reduced quality of life, prolonged hospital stay, and increased hospital costs (2–5). Early nutritional therapy can improve a patient’s prognosis by identifying risk early and moving the timing of intervention forward.

Many tools are available for nutritional risk screening, but at present, there is no gold standard. The Nutritional Risk Score 2002 (NRS2002) is a nutritional screening tool launched by the European Society for Clinical Nutrition and Metabolism (ESPEN) in 2002 and applicable to adult inpatients (6). It is based on 128 randomized controlled clinical studies. The Mini Nutritional Assessment – Short Form (MNA-SF) and the Geriatric Nutritional Risk Index (GNRI) have been validated for the diagnosis of malnutrition and prediction of clinical outcomes in elderly patients (7, 8). The Prognostic Nutritional Index (PNI) is an indicator used to assess the nutritional status of surgical patients, to predict the risk of surgery and to make prognostic judgments (9). The four nutritional screening tools mentioned above can all be used for geriatric surgical patients. However, no studies comparing the effectiveness as a prognostic indicator of these four tools in geriatric surgical patients have been published.

The aim of this study was to explore the correlation between the results of the above-mentioned nutritional risk screening tools and the prognosis in elderly surgical patients, using orthopedic and neurosurgical patients as examples to provide a reference for the selection of appropriate nutritional risk screening tools in clinical practice to more accurately identify high-risk groups before surgery and implement early and effective interventions.

## 2. Materials and methods

### 2.1. Study design and setting

This study is a single-center observational study from January 2021 to March 2022. Subjects were elderly patients who were admitted to the orthopedic or neurosurgical department of West China Hospital, Sichuan University (WCH) for surgical treatment. The study was approved by the Ethics Committee of WCH (2020/1178).

### 2.2. Study population

The following criteria were used to determine inclusion: (1) age ≥ 60; (2) planned surgical treatment; and (3) informed consent to participate in this study. Exclusion criteria were as follows: (1) patients undergoing day surgery or emergency surgery; (2) implantation of pacemakers or other metal implants; (3) end-stage disease (advanced malignant tumor patients, brain death or other end-stage patients); (4) communication disorders such as severe hearing impairment or cognitive impairment; and (5) participation in other nutrition-related research projects.

### 2.3. Data collection

#### 2.3.1. Measurements

A questionnaire was used to collect general information, including sex, age, height, body weight, mental status, Charlson comorbidity index, etc. after admission and before surgery. Body weight was collected at admission. Body composition indexes were collected from patients by means of an Inbody S10 Biospace multifrequency bioelectrical impedance body composition analyzer. Body mass index (BMI), skeletal muscle mass index (SMI), body fat percentage (BFP), arm muscle circumference (AMC) and waist circumference (WC) were collected and analyzed. Clinical data (diagnosis, comorbidity, laboratory test, LOS and postoperative complications, etc.) during hospitalization were collected from the electronic medical records system. Nutrition-related laboratory tests included hemoglobin (HB), serum albumin (ALB), and lymphocyte-monocyte ratio (LMR).

#### 2.3.2. Nutritional screening tool

Four screening tools (MNA-SF, NRS2002, PNI and GNRI) were used to assess elderly orthopedic and neurosurgical patients’ baseline nutritional status. The NRS2002 was performed by nurses within 24 h of admission, while the other three assessments were performed by a clinical dietitian within two days after admission and before surgery. Patients were divided into two categories according to each screening tool and various screening tools were compared.

The NRS-2002 is calculated from BMI, age, weight loss, recent decrease in food intake and disease severity (6), and is suitable for adult inpatients aged 18-90 years old. The total score is 6 points, and is determined by three components: severity of illness score (0-3 points), nutritional status score (0-3 points), and age score (0-1 points). A score of ≥ 3 indicates nutritional risk.

There are six items totaling 14 points on the MNA-SF developed for the elderly: a decrease in food intake in the previous three months, involuntary weight loss in the previous three months, mobility, psychological stress or acute disease in the previous three months, neuropsychological problems, and BMI or calf circumference (if data on the BMI was not available, as in bedridden or comatose patients) (7). Based on the total score, patients were divided into two categories: 12-14 points indicating ‘well-nourished’, and ≤ 11 points indicating ‘malnourished or at risk’.

The PNI and GNRI are objective screening tools that assess the nutritional status and predict patient prognosis. The former is calculated by serum albumin and lymphocytes: PNI = albumin (g/L) + 5 × lymphocyte count (×10^9^/L). Referring to previous studies (9), a cut-off value of 45 was used in this study, indicating ≥ 45 points as ‘well-nourished’, and < 45 points as ‘malnourished’. The formula for GNRI was as follows (8): GNRI = (1.489 × albumin (g/l) + 41.7 × weight (kg)/ideal body weight (kg)). The ideal body weight was calculated by the Lorentz equation, which for women and men was calculated differently. If weight/ideal body weight was ≥ 1.0, it was set to 1. In this study, only two classes were used instead of the standard four classes: namely no risk (GNRI > 98) and at risk (GNRI ≤ 98).

#### 2.3.3. Follow-up

Patients were followed up by telephone one month after surgery. Follow-up visits included the occurrence of surgery-related complications, mainly including infections, major adverse cardiovascular events, status epilepticus and persistent gastrointestinal discomfort (e.g. vomiting, diarrhea).

### 2.4. Statistical analysis

Descriptive characteristics for categorical and continuous variables are presented. Continuous parametric data are expressed as the mean (±standard deviation), while continuous non-parametric data are represented by the median (25th–75th percentile). Frequencies and percentages were used to summarize categorical variables.

Comparisons between groups (well-nourished/no risk and malnourished/at risk) were made using a Student’s t test for continuous parametric variables, the Mann–Whitney test for non-parametric variables, and the chi-squared (X^2^) test for categorical variables. The association between nutritional risk classifications and complications, and LOS was analyzed by using logistic regression analysis adjusted for potential confounding factors (age, sex, lying in bed on admission, and the Charlson comorbidity index). LOS over 7 (median) days for orthopedic patients and LOS over 8 (median) days for neurosurgical patients were considered prolonged hospital stays. All tests were two-sided, and a P-value of < 0.05 was regarded as statistically significant. IBM SPSS Statistics 19.0 was used for statistical analysis.

## 3. Results

During the research phase, a total of 217 elderly perioperative orthopedic patients and 114 elderly perioperative neurosurgical patients met the inclusion criteria. Among them, 45 patients did not undergo body composition analysis preoperatively, 3 patients did not undergo MNA-SF, and 12 patients did not undergo NRS2002. Finally, 167 orthopedic patients and 103 neurosurgical patients were analyzed in this study. Patient characteristics are shown in Table 1. The average age of orthopedic patients was 70.40 years (range: 60-90 years), and women accounted for 72.5% of all participants. The majority of patients was admitted for surgery due to fractures or osteoarthritis. The average age of neurosurgical patients was 67.63 years (range: 60-85 years), and both genders accounted for nearly half of the total participants. Neurological tumors were found in 82.5% of all patients.

**TABLE 1:** Characteristics of participants.

Variables	Orthopedic patients (*n* = 167)	Neurosurgical patients (*n* = 103)
Age, years	70.40 ± 6.52	67.63 ± 4.96
**Sex, n (%)**		
Male	46 (27.5)	51 (49.5)
Female	121 (72.5)	52 (50.5)
Lie in bed on admission, n (%)	39 (23.4)	19 (18.4)
**Disease type**		
Trauma	67 (40.1)	6 (5.8)
Tumor	1 (0.6)	85 (82.5)
Arthropathy	96 (57.5)	/
Discopathy	/	4 (3.9)
Others	3 (1.8)	8 (7.8)
**Charlson comorbidity index**		
0	103 (61.7)	34 (33.0)
1	44 (26.3)	10 (9.7)
>1	20 (12.0)	59 (57.3)
Length of hospital stay, days	7 (5-9)	8 (7-11)

Patients were assessed by four nutritional screening tools and divided into two categories (Table 2). 14 (8.4%) and 21 (12.6%) of the elderly orthopedic patients were malnourished or at risk of malnutrition, respectively, according to NRS2002 and PNI. However the rate of being malnourished or at risk of malnutrition was higher based on the MNA-SF (26.4%) or the GNRI (24.6%), and only 4 (3.9%) of the elderly neurosurgical patients were at risk when evaluated by PNI. According to NRS2002 and PNI, the rates of old neurosurgical patients who were malnourished or at risk of malnutrition were 14.6 and 3.9%, respectively, which was less than the results assessed by the MNA-SF (24.3%) and GNRI (15.5%).

**TABLE 2:** Nutritional status or risk of patients assessed by nutritional screening tools.

Patients	Nutritional status/Risk	MNA-SF (%)	NRS2002 (%)	PNI (%)	GNRI (%)
Orthopedic patients	Well-nourished/no risk	123 (73.7%)	153 (91.6%)	146 (87.4%)	126 (75.4%)
	Malnourished/at risk	44 (26.4%)	14 (8.4%)	21 (12.6%)	41 (24.6%)
Neurosurgical patients	Well-nourished/no risk	78 (75.7%)	88 (85.4%)	99 (96.1%)	87 (84.5%)
	Malnourished/at risk	25 (24.3%)	15 (14.6%)	4 (3.9%)	16 (15.5%)

MNA-SF, Mini Nutritional Assessment-Short Form; NRS2002, Nutrition Risk Screening 2002; PNI, Prognostic Nutritional Index; GNRI, Geriatric Nutritional Risk Index.

The variations in classical nutritional markers were compared between the two groups divided by four nutritional screening tools (Tables 3-6). When applied to orthopedic patients, and compared with the well-nourished/no risk group, the malnourished/at-risk group recognized by NRS2002, PNI or GRNI had a significantly lower ALB, HB and LMR (*P* < 0.05). However, no significant differences in LMR were observed between the two groups classified by MNA-SF. When categorized by NRS2002, no differences were observed in any body composition indicators between no risk and at-risk elderly orthopedic patients. Morever, when categorized by PNI, only an association between SMI and nutritional status was found (*P* = 0.044). The well-nourished group, as determined by the MNA-SF, had a greater BMI, SMI, and WC (*P* < 0.001) Whereas, according to the GNRI, differences in BMI, SMI, BFP and AMC were identified between no risk and at-risk groups (*P* < 0.01).

**TABLE 3:** Relationship between nutritional status or risk assessed by four nutritional screening tools and traditional nutrition laboratory tests (orthopedic patients).

Nutritional status/Risk	ALB (g/L)		HB (g/L)		LMR	
	** $\bar{x}\pm s$ **	***P**-* **value**	** $\bar{x}\pm s$ **	***P**-* **value**	** $\bar{x}\pm s$ **	***P**-* **value**
**MNA-SF**						
Well-nourished	43.20 ± 4.70	<0.001	130.28 ± 16.01	<0.001	3.76 ± 1.53	0.101
Malnourished/At risk	39.98 ± 4.70		115.66 ± 18.11		3.13 ± 2.35	
**NRS2002**						
No risk	42.68 ± 4.67	0.004	127.90 ± 16.22	0.023	3.68 ± 1.82	0.049
At risk	38.74 ± 6.02		110.29 ± 25.33		2.69 ± 1.22	
**PNI**						
Well-nourished	43.65 ± 3.54	<0.001	129.45 ± 15.89	<0.001	3.92 ± 1.69	<0.001
Malnourished	33.32 ± 3.18		105.42 ± 15.93		1.38 ± 0.52	
**GNRI**						
No risk	44.49 ± 2.96	<0.001	131.95 ± 13.88	<0.001	4.09 ± 1.71	<0.001
At risk	35.78 ± 3.69		109.44 ± 17.69		2.08 ± 1.08	

MNA-SF, Mini Nutritional Assessment-Short Form; NRS2002, Nutrition Risk Screening 2002; PNI, Prognostic Nutritional Index; GNRI, Geriatric Nutritional Risk Index; ALB, albumin; HB, hemoglobin; LMR, lymphocyte-monocyte ratio.

**TABLE 4:** Association between nutritional status or risk assessed by four nutritional screening tools and body composition (orthopedic patients).

Nutritional status/Risk	BMI (kg/m^2^)		SMI (kg/m^2^)		BFP (%)		AMC (cm)		WC (cm)	
	** $\bar{\text{x}}\pm \text{s}$ **	***P**-* **value**	** $\bar{\text{x}}\pm \text{s}$ **	***P**-* **value**	** $\bar{\text{x}}\pm \text{s}$ **	***P**-* **value**	** $\bar{\text{x}}\pm \text{s}$ **	***P**-* **value**	** $\bar{\text{x}}\pm \text{s}$ **	***P**-* **value**
**MNA-SF**										
Well-nourished	25.53 ± 3.58	<0.001	6.68 ± 1.26	<0.001	45.65 ± 18.59	0.291	25.76 ± 3.08	0.052	86.06 ± 11.15	<0.001
Malnourished/At risk	21.42 ± 3.53		5.76 ± 0.93		42.16 ± 19.24		24.41 ± 5.66		79.28 ± 8.67	
**NRS2002**										
No risk	24.71 ± 3.70	0.067	6.45 ± 1.17	0.764	45.20 ± 18.85	0.286	25.50 ± 3.94	0.271	84.65 ± 10.91	0.144
At risk	21.57 ± 5.80		6.29 ± 1.99		39.59 ± 17.71		24.29 ± 4.06		80.18 ± 10.90	
**PNI**										
Well-nourished	24.61 ± 3.81	0.167	6.511 ± 1.24	0.044	44.86 ± 17.90	0.809	25.57 ± 4.13	0.143	84.52 ± 10.90	0.448
Malnourished	23.32 ± 5.08		5.924 ± 1.24		43.80 ± 24.47		24.22 ± 2.12		82.58 ± 11.42	
**GNRI**										
No risk	25.24 ± 3.36	<0.001	6.57 ± 1.24	0.001	46.49 ± 17.38	0.033	25.89 ± 4.25	0.005	85.82 ± 10.89	0.165
At risk	22.02 ± 4.78		6.02 ± 1.21		39.31 ± 21.86		23.92 ± 2.31		79.53 ± 9.80	

MNA-SF, Mini Nutritional Assessment-Short Form; NRS2002, Nutrition Risk Screening 2002; PNI, Prognostic Nutritional Index; GNRI, Geriatric Nutritional Risk Index; BMI, body mass index; SMI, skeletal muscle mass index; BFP, body fat percentage; AMC, arm muscle circumference; WC, waist circumference.

**TABLE 5:** Relationship between nutritional status or risk assessed by four nutritional screening tools and traditional nutrition laboratory tests (neurosurgical patients).

Nutritional status/Risk	ALB (g/L)		HB (g/L)		LMR	
	** $\bar{\text{x}}\pm \text{s}$ **	***P**-* **value**	** $\bar{\text{x}}\pm \text{s}$ **	***P**-* **value**	** $\bar{\text{x}}\pm \text{s}$ **	***P**-* **value**
**MNA-SF**						
Well-nourished	42.57 ± 2.92	0.030	133.47 ± 12.98	0.023	3.77 ± 1.44	0.189
Malnourished/At risk	41.07 ± 3.03		125.88 ± 17.87		3.34 ± 1.42	
**NRS2002**						
No risk	42.39 ± 2.97	0.120	132.03 ± 13.40	0.500	3.68 ± 1.47	0.806
At risk	41.09 ± 3.07		129.27 ± 20.72		3.58 ± 1.25	
**PNI**						
Well-nourished	42.45 ± 2.78	<0.001	132.06 ± 14.64	0.138	3.76 ± 1.38	0.001
Malnourished	36.00 ± 0.95		121.00 ± 9.13		1.34 ± 0.29	
**GNRI**						
No risk	43.00 ± 2.36	<0.001	133.70 ± 13.10	0.001	3.82 ± 1.38	0.014
At risk	37.85 ± 2.35		120.38 ± 17.43		2.87 ± 1.53	

MNA-SF, Mini Nutritional Assessment-Short Form; NRS2002, Nutrition Risk Screening 2002; PNI, Prognostic Nutritional Index; GNRI, Geriatric Nutritional Risk Index; ALB, albumin; HB, hemoglobin; LMR, lymphocyte-monocyte ratio.

**TABLE 6:** Association between nutritional status or risk assessed by four nutritional screening tools and body composition (neurosurgical patients).

Nutritional status/Risk	BMI (kg/m^2^)		SMI (kg/m^2^)		BFP (%)		AMC (cm)		WC (cm)	
	** $\bar{\text{x}}\pm \text{s}$ **	***P**-* **value**	** $\bar{\text{x}}\pm \text{s}$ **	***P**-* **value**	** $\bar{\text{x}}\pm \text{s}$ **	***P**-* **value**	** $\bar{\text{x}}\pm \text{s}$ **	***P**-* **value**	** $\bar{\text{x}}\pm \text{s}$ **	***P**-* **value**
**MNA-SF**										
Well-nourished	24.09 ± 2.84	0.002	6.73 ± 0.99	0.040	45.71 ± 14.40	0.611	25.86 ± 2.73	0.006	84.72 ± 9.33	0.074
Malnourished/At risk	21.65 ± 4.43		6.25 ± 1.05		44.03 ± 14.08		24.18 ± 2.14		80.60 ± 11.62	
**NRS2002**										
No risk	23.82 ± 3.35	0.020	6.68 ± 1.03	0.097	45.71 ± 14.04	0.489	25.60 ± 2.74	0.175	84.26 ± 10.03	0.193
At risk	21.61 ± 3.45		6.21 ± 0.85		42.93 ± 15.89		24.58 ± 2.30		80.60 ± 9.77	
**PNI**										
Well-nourished	23.55 ± 3.48	0.489	6.60 ± 1.02	0.705	45.55 ± 14.40	0.383	25.48 ± 2.73	0.691	83.85 ± 10.07	0.518
Malnourished	22.33 ± 2.25		6.80 ± 1.03		39.17 ± 9.98		24.93 ± 1.26		80.53 ± 9.51	
**GNRI**										
No risk	24.09 ± 3.28	<0.001	6.72 ± 1.01	0.012	45.75 ± 13.93	0.457	25.79 ± 2.72	0.002	85.01 ± 9.76	0.002
At risk	20.67 ± 3.15		6.03 ± 0.84		42.85 ± 16.31		23.60 ± 1.59		76.74 ± 8.74	

MNA-SF, Mini Nutritional Assessment-Short Form; NRS2002, Nutrition Risk Screening 2002; PNI, Prognostic Nutritional Index; GNRI, Geriatric Nutritional Risk Index; BMI, body mass index; SMI, skeletal muscle mass index; BFP, body fat percentage; AMC, arm muscle circumference; WC, waist circumference.

For neurosurgical patients, no differences in any laboratory indicators were observed between no risk and at-risk elderly patients when classified by NRS2002. When categorized by the MNA-SF, the well-nourished group showed higher level of ALB and HB (*P* < 0.05), while the LMR did not differ from the malnourished/at risk group. Furthermore, when categorized by PNI, significant differences were observed in ALB (*P* < 0.001) and LMR (*P* = 0.001). Only when the GNRI screening method was used, differences in all laboratory indicators were observed: the at-risk group had lower levels of ALB, HB and LMR (*P* < 0.05). In terms of body composition, none of the screening tools identified between-group differences in percentage of body fat. No differences were observed in any of the body composition indicators between well-nourished and malnourished elderly neurosurgical patients when categorized by PNI. Based on the MNA-SF, the well-nourished group had a higher BMI, SMI, and AMC (*P* < 0.05). Categorized by NRS2002, significant differences was found in BMI (*P* = 0.02), but not in SMI, AMC, or WC. According to the GNRI, the at-risk group had a lower BMI, SMI, AMC, and WC than the no risk group (*P* < 0.01).

The association between nutritional status or risk assessed by four nutritional screening tools and length of stay or complication was shown in Tables 7, 8. For elderly orthopedic patients, the nutritional risks shown by the four tools were all associated with the LOS (*P* < 0.05). Only the nutritional risks shown by PNI and GNRI screening were associated with total complications within one month after surgery(*P* < 0.05), The nutritional risks shown by MNA-SF, PNI and GNRI screening were associated with infectious complications (*P* < 0.05). However, the nutritional risk shown by the four screening tools was not significantly associated with preoperative hospital stay, total complications, infectious complications or non-infectious complications at 1 month in elderly patients undergoing neurosurgery, but the nutritional risk shown by PNI and GNRI screening was associated with overall hospital stay and postoperative hospital stay (*P* < 0.01).

**TABLE 7:** Associations between nutritional status or risk assessed by four nutritional screening tools and length of hospital stay or complication (orthopaedics patients).

Nutritional status/risk	LOS		Preoperative LOS		Postoperative LOS		Complication		Infectious complication		Non-infectious complication	
	**Median (IQR)**	***P**-* **value**	**Median (IQR)**	***P**-* **value**	**Median (IQR)**	***P**-* **value**	***N*** **(%)**	***P**-* **value**	***N*** **(%)**	***P**-* **value**	***N*** **(%)**	***P**-* **value**
**MNA-SF**												
Well-nourished	6 (5-8)	<0.001	3 (2-4)	0.002	3 (3-4)	0.001	8 (6.50)	0.118^a^	6 (4.88)	0.044^a^	3 (2.44)	1.000^b^
Malnourished/At risk	9 (5-13)		4 (3-5)		5 (3-7)		7 (15.91)		7 (15.91)		1 (2.27)	
**NRS2002**												
No risk	6 (5-9)	0.001	3 (2-4)	0.001	3 (3-4)	< 0.001	12 (7.84)	0.225^a^	10 (6.54)	0.142^a^	4 (2.61)	1.000^b^
At risk	12 (8-18)		6 (3-8)		7 (4-10)		3 (21.43)		3 (21.43)		0 (0.00)	
**PNI**												
Well-nourished	6 (5-8)	< 0.001	3 (2-4)	< 0.001	3 (3-5)	0.005	9 (6.16)	0.003^a^	8 (5.48)	0.013^a^	2 (1.37)	0.078^b^
Malnourished	10 (8-12)		4 (4-7)		4 (4-6)		6 (28.57)		5 (23.81)		2 (9.52)	
**GNRI**												
No risk	6 (5-8)	< 0.001	3 (2-4)	< 0.001	3 (3-4)	< 0.001	7 (5.56)	0.016^a^	6 (4.76)	0.026^a^	2 (1.37)	0.253^b^
At risk	10 (8-13)		4 (3-7)		4 (3-7)		8 (19.51)		7 (17.07)		2 (9.52)	

MNA-SF, Mini Nutritional Assessment-Short Form; NRS2002, Nutrition Risk Screening 2002; PNI, Prognostic Nutritional Index; GNRI, Geriatric Nutritional Risk Index; IQR, interquartile range; LOS, lengths of stay.

^a^Continuity correction.

**TABLE 8:** Association between nutritional status or risk assessed by four nutritional screening tools and length of hospital stay or complication (neurosurgical patients).

Nutritional status/Risk	LOS		Preoperative LOS		Postoperative LOS		Complication		Infectious complication		Non-infectious complication	
	**Median (IQR)**	***P**-* **value**	**Median (IQR)**	***P**-* **value**	**Median (IQR)**	***P**-* **value**	***N*** **(%)**	***P**-* **value**	***N*** **(%)**	***P**-* **value**	***N*** **(%)**	***P**-* **value**
**MNA-SF**												
Well-nourished	8 (7-11)	0.306	3 (2-4)	0.485	6 (4-6)	0.062	11 (14.10)	1.000^a^	7 (8.97)	0.955^a^	5 (6.41)	1.000^b^
Malnourished/At risk	8 (8-19)		2 (2-4)		6 (5-11)		4 (16.00)		3 (12.00)		1 (4.00)	
**NRS2002**												
No risk	8 (7-11)	0.876	2 (2-4)	0.909	6 (4-7)	0.860	14 (15.91)	0.588^a^	9 (10.23)	1.000^a^	6 (6.82)	0.589^b^
At risk	8 (7-11)		2 (2-5)		6 (4-7)		1 (6.67)		1 (6.67)		0 (0.00)	
**PNI**												
Well-nourished	8 (7-11)	< 0.001	2 (2-4)	0.065	6 (4-6)	0.002	14 (14.14)	0.548^b^	9 (9.09)	0.340^b^	6 (6.06)	1.000^b^
Malnourished	22 (15-38)		7 (3-8)		15 (8-34)		1 (25.00)		1 (25.00)		0 (0.00)	
**GNRI**												
No risk	8 (7-10)	0.004	2 (2-4)	0.954	6 (4-6)	< 0.001	10(11.49)	0.094^a^	6 (6.90)	0.074^a^	5 (5.75)	1.000^b^
At risk	12 (8-25)		3 (2-6)		8 (6-20)		5 (16.00)		4 (25.00)		1 (6.25)	

MNA-SF, Mini Nutritional Assessment-Short Form; NRS2002, Nutrition Risk Screening 2002; PNI, Prognostic Nutritional Index; GNRI, Geriatric Nutritional Risk Index; IQR, interquartile range; LOS, lengths of stay.

^a^Continuity correction.

^b^Fisher’s exact test.

The results of the logistic regression analyses for orthopedic patients are shown in Table 9. After controlling for potential confounders, a significant relationship between the PNI (malnourished vs. well-nourished, OR = 5.39, 95% CI: 1.11-26.18, *P* = 0.037), GNRI (at risk vs. no risk, OR = 3.96, 95% CI: 1.01-15.45, *P* = 0.048) and complications remained. For infectious complications, associations were only observed with GNRI (at risk vs. no risk, OR = 4.38, 95% CI: 1.03-18.63, *P* = 0.046). Nonetheless, after multivariate analysis, Only GNRI was significantly related to LOS > 7 days (at risk vs. no risk, OR = 4.01, 95% CI: 1.64-9.80, *P* = 0.002). Table 10 shows the results of the regression analysis performed on neurosurgical patients. In the multivariate model, none of the four tools was relevant for the complications. Similarly, an association between GNRI and LOS > 8 days was discovered in neurosurgical patients (at risk vs. no risk, OR = 3.35, 95% CI: 1.03-10.86, *P* = 0.002).

**TABLE 9:** Adjusted and unadjusted analysis of the association between complications, length of stay and each nutritional status at admission by the four nutritional screening tools (orthopedic patients).

Nutritional status/Risk	Complication				Infectious complication				Non-infectious complication				LOS > 7 (day)			
	**Unadjusted OR (95% CI)**	***P**-* **value**	**Adjusted OR^Ψ^ (95% CI)**	***P**-* **value**	**Unadjusted OR (95% CI)**	***P**-* **value**	**Adjusted OR^Ψ^(95% CI)**	***P**-* **value**	**Unadjusted OR (95% CI)**	***P**-* **value**	**Adjusted OR^Ψ^(95% CI)**	***P**-* **value**	**Unadjusted OR (95% CI)**	***P**-* **value**	**Adjusted OR^Ψ^(95% CI)**	***P**-* **value**
**MNA-SF**																
Well-nourished	References															
Malnourished/At risk	2.72 (0.92, 8.01)	0.069	1.85 (0.53, 6.41)	0.334	3.69 (1.17, 11.67)	0.026	2.50 (0.68, 9.20)	0.169	0.93 (0.09, 9.18)	0.951	0.87 (0.06, 12.18)	0.916	3.50 (1.70, 7.19)	0.001	2.21 (0.99, 4.96)	0.054
**NRS2002**																
No risk	References															
At risk	3.21 (0.79, 13.07)	0.105	3.08 (0.56, 16.97)	0.197	3.90 (0.94, 16.27)	0.062	4.03 (0.71, 22.92)	0.116	0.00 (0.00, 0.00)	0.999	0.00 (0.00, 0.00)	0.998	6.01 (1.60, 22.43)	< 0.001	3.791 (0.87.16.46)	0.075
**PNI**																
Well-nourished	References															
Malnourished	6.09 (1.90, 19.47)	0.002	5.39 (1.11, 26.18)	0.037	5.39 (1.57, 18.47)	0.007	5.09 (0.97, 26.82)	0.055	7.58 (1.01, 57.00)	0.049	4.71 (0.19, 115.09)	0.342	5.62 (1.95, 16.20)	0.001	2.232 (0.65, 7.65)	0.202
**GNRI**																
No risk	References															
At risk	4.12 (1.39, 12.20)	0.011	3.96 (1.01, 15.45)	0.048	4.12 (1.30, 13.07)	0.016	4.38 (1.03, 18.63)	0.046	3.18 (0.43, 23.32)	0.255	0.992 (0.052, 18.84)	0.996	7.18 (3.20, 16.11)	< 0.001	4.01 (1.64. 9.80)	0.002

MNA-SF, Mini Nutritional Assessment-Short Form; NRS2002, Nutrition Risk Screening 2002; PNI, Prognostic Nutritional Index; GNRI, Geriatric Nutritional Risk Index; LOS, lengths of stay.

^Ψ^Adjusted for age, sex, lie in bed on admission, and charlson comorbidity index.

**TABLE 10:** Adjusted and unadjusted analysis of the association between complications, length of stay and each nutritional status at admission by the four nutritional screening tools (neurosurgical patients).

Nutritional status/Risk	Complication				Infectious complication				Non-infectious complication				LOS > 8 (day)			
	**Unadjusted OR (95% CI)**	***P***-**value**	**Adjusted OR^Ψ^(95% CI)**	***P***-**value**	**Unadjusted OR (95% CI)**	***P***-**value**	**Adjusted OR^Ψ^(95% CI)**	***P***-**value**	**Unadjusted OR (95% CI)**	***P***-**value**	**Adjusted OR^Ψ^(95% CI)**	***P***-**value**	**Unadjusted OR (95% CI)**	***P***-**value**	**Adjusted OR^Ψ^(95% CI)**	***P***-**value**
**MNA-SF**																
Well-nourished	References															
Malnourished/At risk	1.16 (0.33, 4.03)	0.815	0.66 (0.16, 2.81)	0.575	1.383 (0.33,5.81)	0.658	0.705 (0.13, 3.84)	0.686	0.61 (0.07, 5.47)	0.657	0.73 (0.07, 7.58)	0.795	1.07 (0.43, 2.66)	0.882	0.76 (0.27, 2.09)	0.590
**NRS2002**																
No risk	References															
At risk	0.38 (0.05, 3.11)	0.365	0.27 (0.03, 2.39)	0.239	0.63 (0.07, 5.34)	0.669	0.42 (0.04, 3.97)	0.488	0.00 (0.00, 0.00)	0.999	0.00 (0.00, 0.00)	0.998	1.21 (0.40, 3.62)	0.738	1.02 (0.33, 3.21)	0.971
**PNI**																
Well-nourished	References															
Malnourished	2.02 (0.20, 20.86)	0.554	1.41 (0.11, 18.62)	0.796	3.33 (0.31, 35.47)	0.318	2.58 (0.19, 35.74)	0.481	0.00 (0.00, 0.00)	0,999	0.00 (0.00, 0.00)	0.999	2.38 (0.00, +∞)	0.999	2.69 (0.00, +∞)	0.999
**GNRI**																
No risk	References															
At risk	3.50 (1.01, 12.16)	0.049	3.14 (0.85, 11.54)	0.085	4.50 (1.11, 18.30)	0.036	3.86 (0.87, 17.14)	0.076	1.09 (0.12,10.03)	0.937	1.22 (0.12, 12.87)	0.869	3.60 (1.15, 11.28)	0.028	3.35 (1.03,10.86)	0.044

MNA-SF, Mini Nutritional Assessment-Short Form; NRS2002, Nutrition Risk Screening 2002; PNI, Prognostic Nutritional Index; GNRI, Geriatric Nutritional Risk Index; LOS, lengths of stay.

^Ψ^Adjusted for age, sex, lie in bed on admission, and charlson comorbidity index.

## 4. Discussion

This study aimed to assess the correlation between nutritional status, evaluated using four nutritional screening tools prior to surgery, and the LOS as well as complications within a month after surgery in elderly patients. Orthopedics and neurosurgery are two important branches of surgery. In this study, patients from these two departments were included as surgical patients sample. All analyses were conducted separately to explore whether there were differences in the applicability of the above-mentioned nutritional screening tools across various types of surgeries. To our knowledge, this is the first study to propose an appropriate nutritional screening tool related to prognosis after comparing NRS2002, MNA-SF, PNI and GNRI in geriatric surgical patients.

The prevalence of malnutrition varied depending on the nutritional screening tools used. Our data showed that nutritional risk rates screened by MNA-SF and GNRI were higher than those screened by NRS2002 and PNI in both orthopedic and neurosurgical elderly populations (Table 2). In a previous study, higher rates of malnutrition were also demonstrated for fracture patients screened with MNA-SF (72.6%) and GNRI (78.5%) compared to those screened with NRS2002 (56.6%) (10). Based on our results, the nutritional risk of elderly orthopedic patients ranged from 8.4 to 26.4%, which was significantly lower than the aforementioned research. This may be attributed to the fact that the nutritional risk of osteoarthropathy patients is much lower than that of fracture patients (10, 11), and arthropathy accounted for 57.5% of the subjects in this study. The core content of the NRS-2002 includes a less sensitive BMI value (12), which makes it more likely to miss some patients whose true nutritional status cannot be judged on the surface but who are actually malnourished. Sarcopenic obesity is also a common finding in the elderly population (13). Mobility, psychological stress and neuropsychological problems were also considered in MNA-SF. Moreover, calf circumference can be used instead of an inaccurate BMI, making MNA-SF more sensitive for elderly individuals and reported highest rate of nutritional risk. Multiple studies of older adults have shown that the nutritional risk rate screening by MNA-SF was higher than NRS2002 (14–17). In our study, the GNRI screened for more malnourished patients possibly because the cut-off values were more appropriate for older patients. In several studies, the cut-off values for PNI were adjusted in elderly adults for different purposes. Panç et al. discovered that the cut-off value of the PNI for 30-day survival after transcatheter aortic valve implantation was 43.37 (18). Xishan et al. classified patients after total gastrectomy into a low PNI group and a high PNI group based on a cut-off value of 43 (19). If we refer to the above studies and take a lower cut-off value, the malnourished rate identified by PNI will be even lower. The data shows that GNRI may have a higher sensitivity than PNI. Among the above tools, groups classified by GNRI showed differences on more classical nutritional markers. However, it was worth mentioning that some of the associations in the tables were to be expected because the parameters associated, featured in the calculation of the relevant tool, such as GNRI and ALB.

Unfortunately, a gold standard for nutritional risk screening and malnutrition has not yet been established. In previous studies, BMI, calf circumference, muscle mass, albumin, the Subjective Global Assessment and the definition of malnutrition proposed by European Society for Clinical Nutrition and Metabolism et al. were used as reference criteria to evaluate the effectiveness of nutritional risk screening tools (20–23). Because not all subjects underwent a professional assessment for nutritional status, the sensitivity, specificity, and negative and positive predictive value of the four nutritional risk screening tools has not been calculated in this study. This is one of the limitations of our study. For further research, we consider the most current Criteria for the Diagnosis of Malnutrition by Global Leadership Initiative on Malnutrition (GLIM) as the semi-gold standard (24). The nutritional screening tools and GLIM Criteria can both be performed in the target population to identify potential screening tools with good sensitivity and specificity (25–28).

In our study, nutritional risk as measured by GNRI was linked to both complications within one month and a longer hospital stay in orthopedic patients. Furthermore, the nutritional risk identified by GNRI in neurosurgical patients was significantly associated with a prolonged hospital stay. Thus, the findings of this study suggested that the GNRI might be the most sensitive tool for predicting complications and prolonged hospital stay in elderly patients undergoing orthopedic and neurosurgical surgery.

The GNRI is a screening tool that is used to predict prognosis in elderly patients and was first proposed in 2005 by Bouillanne et al. (8). The GNRI assesses nutrition-related morbidity and mortality in hospitalized elderly patients using serum albumin and anthropometric data (weight and height). In previous studies, the predictive value of the GNRI was confirmed for postoperative complications (29, 30). In addition, results from a comparable study for hip fracture paitients revealed that both MNA-SF and GNRI were significantly associated with 10-m walking speed, which were not found among the Malnutrition Universal Screening Tool (MUST) and NRS2002 (10). Due to similar results obtained in patients from the two various departments, the GNRI may have a general application value for nutritional risk screening in elderly surgical patients.

Recently, the NRS2002, MNA-SF, Malnutrition Universal Screening Tool (MUST), Malnutrition Screening Tool (MST) and Nutritional Risk Index (NRI) were compared in a Chinese colorectal cancer surgery population (31). Multivariate logistic regression analysis revealed that NRS 2002 ≥ 3 was an independent risk factor for postoperative complications and the strongest predictor of postoperative complications. Research also showed that MNA-SF exhibited a better discrimination than NRS2002 and GNRI in gastrointestinal cancer patients older than 65 years (28). This was not consistent with what was found in patients undergoing non-gastrointestinal surgery. Unfortunately, neither tool showed an association with outcome in older patients. Taken together, these results suggest that there may be differences in the selection of nutritional screening tools between patients undergoing non-gastrointestinal surgery and gastrointestinal surgery.

The concordance between the MNA and GNRI was found to be 39% (32). Both tools showed a significant correlation with anthropometric, biochemical, and Barthel Index scores. As an objective index that does not depend on a caregiver or memory, GNRI was friendly to older patients with cognitive impairment and/or delirium. However, few studies have been published using the GNRI for surgical patients. Therefore, additional studies are needed to verify the validity of GNRI in other types of surgical disease.

Our study has several limitations. First, the sensitivity and specificity of tools have not been calculated based on the “gold standard”. Second, we only included patients from two non-gastrointestinal surgery departments. There should be reservations when applying these findings to emergency surgeries (as well as the gastroenterology surgery as already mentioned). Lastly, limited by the sample size and low complication rate, complications were only categorized into infectious and noninfectious complications. Effects may inconsistencies for more granular categories of complications.

## 5. Conclusion

Even after accounting for confounding factors, the GNRI was found to be significantly associated with the LOS or complications within a month in elderly patients undergoing perioperative orthopedic and neurosurgical procedures. When screening by GNRI, the at-risk group had a longer LOS and a higher complication rate. Thus, we consider that the GNRI is a practical nutritional screening tool to associates with short-term prognosis in elderly perioperative orthopedic and neurosurgical patients. More evidence is needed to confirm the applicability of GNRI in other surgical fields.

## Data availability statement

The original contributions presented in this study are included in the article/supplementary material, further inquiries can be directed to the corresponding authors.

## Ethics statement

The studies involving human participants were reviewed and approved by the Ethics Committee of West China Hospital, Sichuan University. The patients/participants provided their written informed consent to participate in this study. Written informed consent was obtained from the individual(s) for the publication of any potentially identifiable images or data included in this article.

## Author contributions

JG contributed to the study design, data analysis, and manuscript preparation. SZ and JZ participated in nutritional risk screening and questionnaires. JY, LL, and XL performed the body composition analysis and data cleaning. FY and WH were fully involved in the study design and manuscript revision. All authors contributed to the article and approved the submitted version.
